# Biosafety of citrate coated zerovalent iron nanoparticles for Magnetic Resonance Angiography

**DOI:** 10.1016/j.dib.2018.08.157

**Published:** 2018-08-31

**Authors:** Nimi N., Ariya Saraswathy, Shaiju S. Nazeer, Nimmi Francis, Sachin J. Shenoy, Ramapurath S. Jayasree

**Affiliations:** a Division of Biophotonics and Imaging, Biomedical Technology Wing, Sree Chitra Tirunal Institute for Medical Sciences and Technology, Poojappura, Thiruvananthapuram 695012, Kerala, India; b Division of in vivo Models and Testing, Biomedical Technology Wing, Sree Chitra Tirunal Institute for Medical Sciences and Technology, Poojappura, Thiruvananthapuram 695012, Kerala, India

## Abstract

Though nanoparticles are being used for several biomedical applications, the safety of the same is still a concern. It is very routine procedure to check the preliminary safety aspects of the particles intended for *in vivo* applications. The major tests include how the material reacts to a normal cell, how it behaves with the blood cells and also whether any lysis take place in the presence of these materials. Here we present these test data of two novel nanomaterials designed for its use as contrast agent for magnetic resonance imaging and a multimodal contrast agent for targeted liver imaging. On proving the biosafety, the materials were tested for Magnetic Resonance Angiography using normal rats as model. The data of the same were clear identification of the prominent vascular structures and is included as the colour coded MRI image. Lateral and oblique view data are also presented for visualizing other major blood vessels.

**Specifications Table**

**Table:** 

Subject area	*Physics, Chemistry, Biology*
More specific subject area	*Bioimaging*
Type of data	*Image (MRI, Optical, microscopy, etc), text file, graph, figure*
How data was acquired	*MRI (MAGNETOM Avanto Tim System 1.5 T - Siemens, Munich, Germany), IVIS (Xenogen, in vivo imaging system (Caliper Life Sciences, Hopkinton, MA, USA)), DLS ((Malvern Instruments Limited, UK), Microscope (Leica DM IRB, Germany), TEM (TEM, JEM-2010, JEOL, Tokyo, Japan), confocal Raman spectroscopy (Witec alpha 300RA Raman spectrometer),XRD (X’Pert PRO,), FTIR (Thermo Nicolet 5700 FTIR spectrometer (USA)),UV–vis Spectrophotometer (Shimadzu UV Spectrophotometer UV-1800),Fluorescence Spectrophotometer(SPECTRACQ) VSM (SQUID-VSM, Quantum Design). Cell culture etc.*
Data format	*Analysed*
Experimental factors	*The study deals with the synthesis of multimodal contrast agent and their in vitro and in vivo experiments. Pre treatment is not applicable for any of these*
Experimental features	*Citrate coated zerovalent iron nanoparticles was prepared as a novel MRA contrast agent and carbon dots coated pullulan stabilized zerovalent iron nanoparticles were also prepared as a bimodal contrast agent used for liver targeted MRI and optical Imaging.*
Data source location	*Thiruvananthapuram, Kerala, India*
Data accessibility	*The raw/processed data required to reproduce these findings cannot be shared at this time as the data also forms part of an ongoing study.*

**Value of the data**

• The hemolysis, blood compatibility and cytotoxicity data will be of immense use when this novel material, C@ZVI and P@ZVI-Cdts developed for MRA and multimodal imaging, if this material is considered for clinical application, in future.
• The color coded MRA images give a clear view of the vascular structure when the novel positive contrast agent, C@ZVI was used for angiogram. Here also the data of the MRA of an animal using a novel material will be useful to compare with the existing contrast materials in the market and also to extend the research to newer directions which could lead to the development of an ideal MRI contrast agent for angiogram
• The dorsal and lateral oblique view data of the animal MR angiogram identifies pulmonary trunk, pulmonary artery branches and hepatic portal vein. These information will also support the previous ones.

## 1 Data

**Fig. 1:**
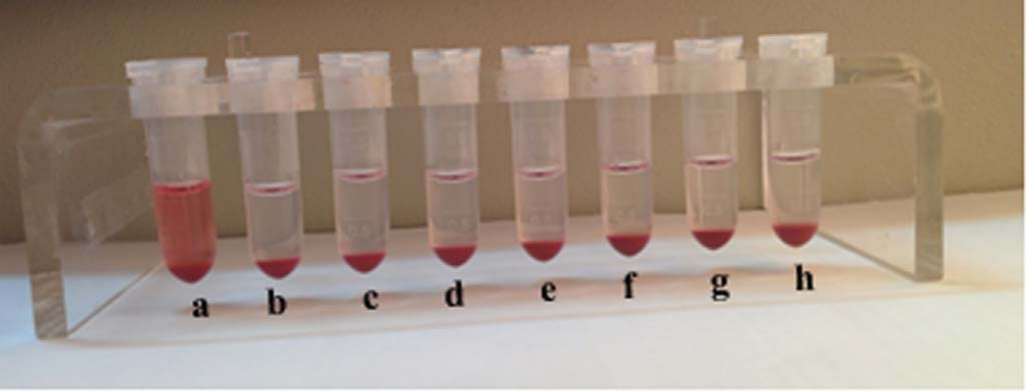
Hemolysis study: (a)Water, positive control (b–d) different concentrations (50,100,200 mM) of C@ZVI (e–g) different concentrations (50,100,200 mM) of P@ZVI-Cdts and (h) Cdts.

The data of hemolysis analysis of C@ZVI, P@ZVI-Cdts and Cdts are shown in Fig. 1.

**Fig. 2:**
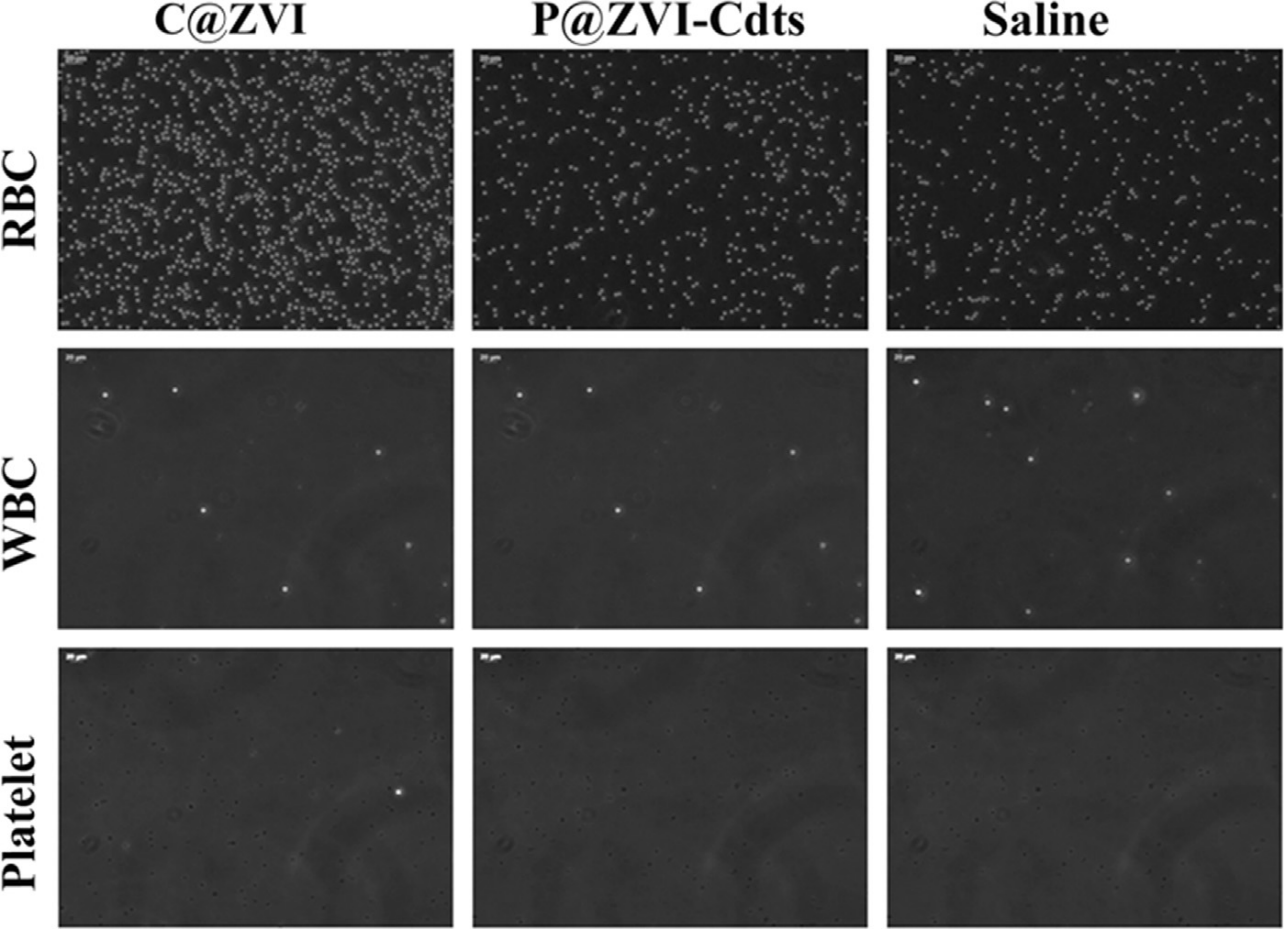
Blood aggregation study: Phase contrast images (20×) of RBC, WBC and platelet incubated with 200 µg of C@ZVI and P@ZVI-Cdts respectively, along with saline as control.

**Fig. 3:**
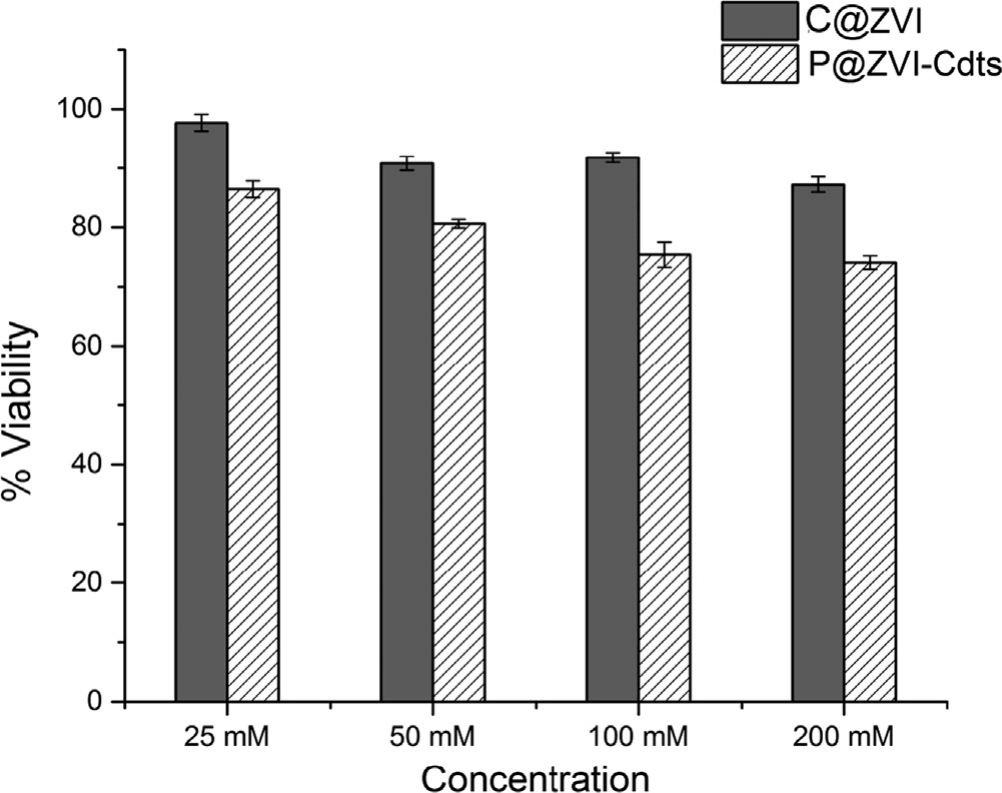
Percentage viability of L929 cells on treatment with different concentrations of C@ZVI and P@ZVI-Cdts, by MTT assay.

**Fig. 4:**
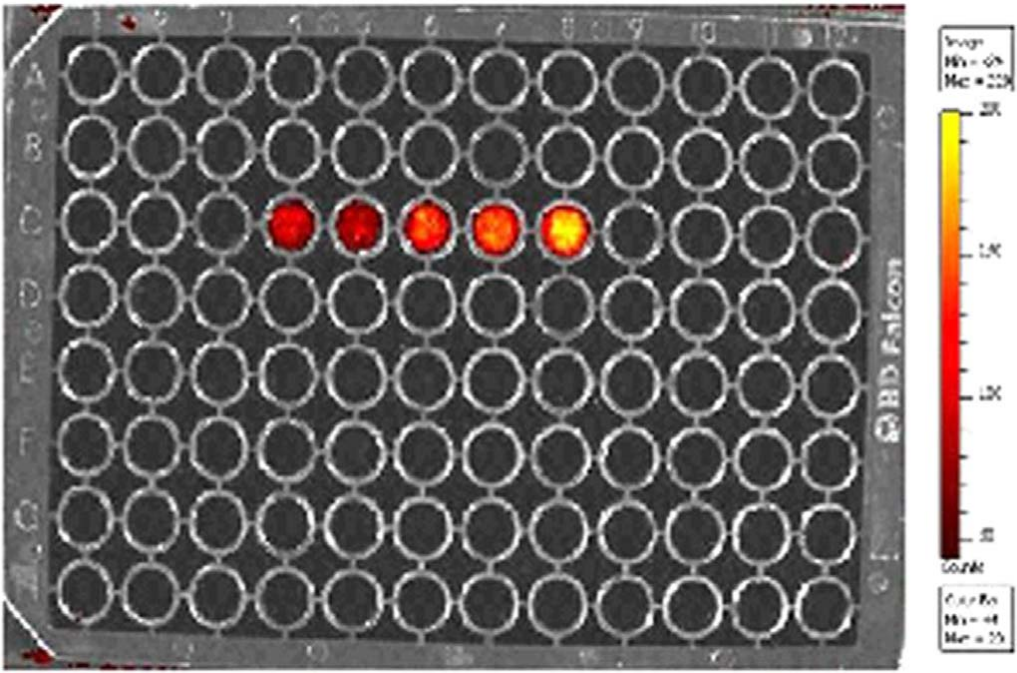
Fluorescence imaging intensity at different concentrations (50, 100, 200, 250 mM) of P@ZVI-Cdts using IVIS Imaging system.

**Fig. 5:**
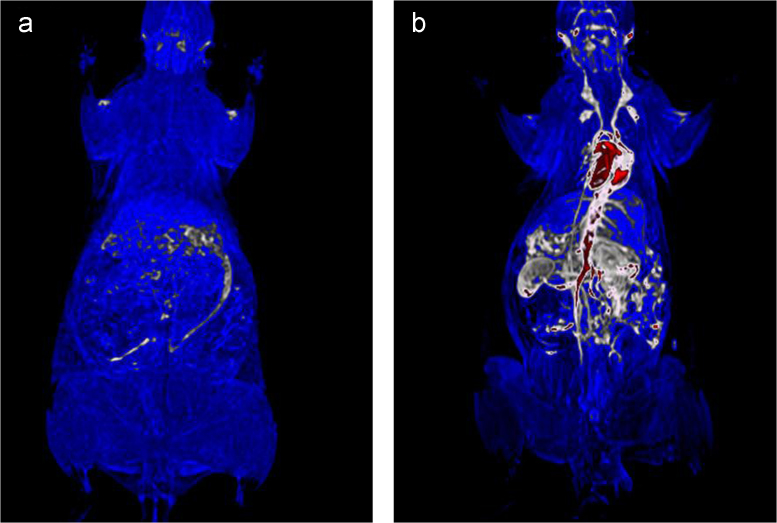
Color code images of C@ZVI enhanced Magnetic Resonance Angiography (MRA) on rat model (a) Control animal (b) C@ZVI administrated animal.

**Fig. 6:**
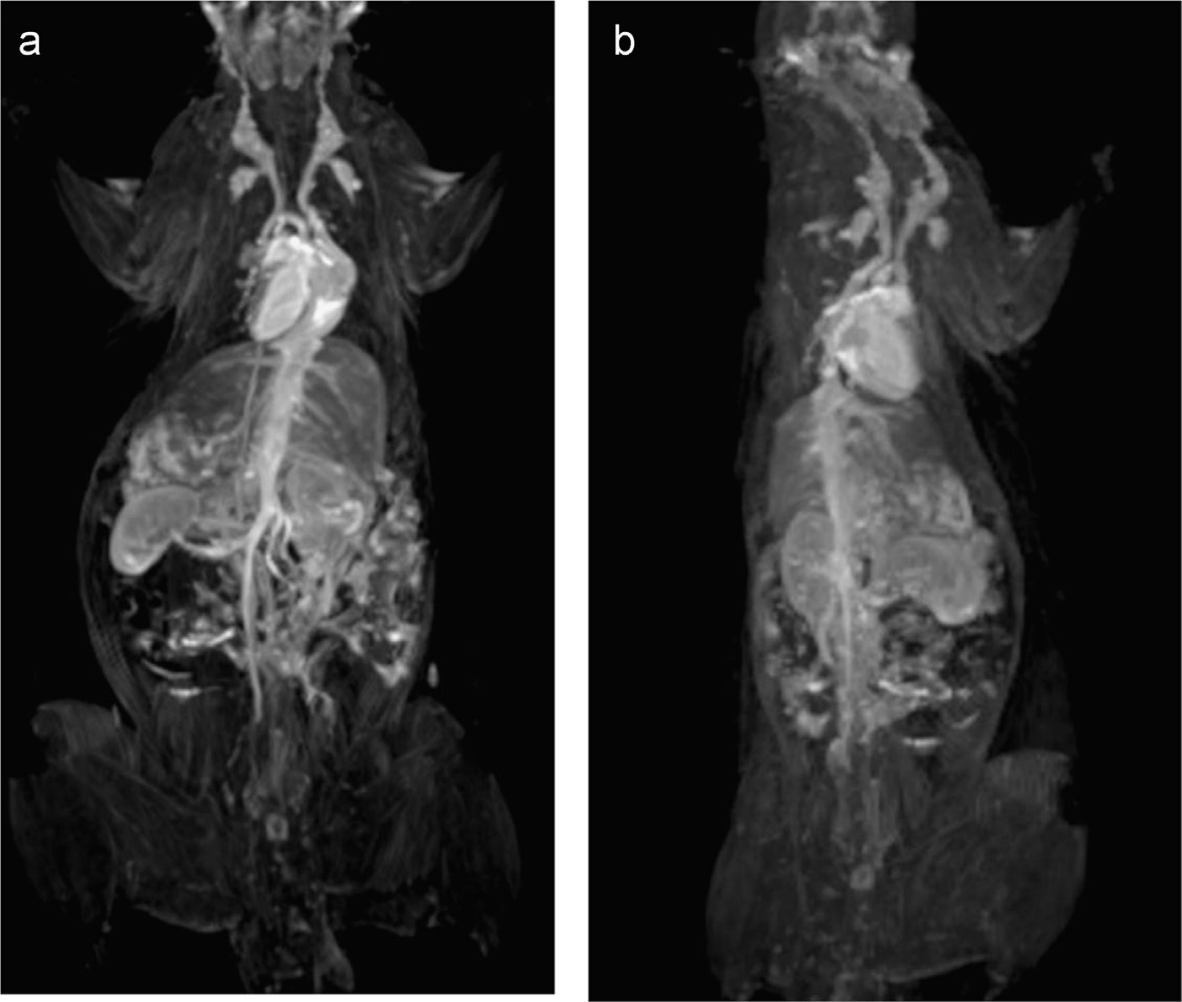
The oblique views of C@ZVI enhanced Magnetic Resonance Angiography (MRA) on rat model (a) dorsal oblique (b) lateral oblique.

Blood cell aggregation study data are provided in Fig. 2 and the cytotoxicity study data using normal fibroblast cells,L929 is given in Fig. 3. The fluorescence imaging data of P@ZVI-Cdts acquired using an optical imaging system is shown in Fig. 4. The colour coded MRA data and the dorsal and lateral oblique view data of Magnetic resonance imaging to view the blood vessels are shown in Fig. 5, Fig. 6.

## 2 Experimental design, materials and methods

### 2.1 Blood compatibility studies

The samples were checked for their blood compatibility by examining the hemolysis and the blood cell aggregation response. For hemolysis assay, human blood collected from volunteers was centrifuged at 700RPM for 10 min. The RBC thus obtained was washed and diluted with saline. Then 100 µl of C@ZVI nanoparticles and P@ZVI-Cdts at different concentrations (50,100, 200 mM) were dispersed in saline and was added to 100 µl of washed RBC and mixed gently and incubated for 2 h at 37 °C. The supernatant was collected and read at 541 nm by UV spectrophotometer (Carywin UV). Saline was used as the negative control and distilled water as a positive control. The RBC, WBC, and platelet aggregation study were carried out to rule out the aggregation of the blood cells when in contact with a foreign body for intravenous injection when used as a contrast agent, as well as for optical imaging *in vivo*. For this, 200 µg of the C@ZVI nanoparticles and P@ZVI-Cdts, were added to 100 µl of the diluted blood cells and incubated for 30 min at 37 °C. Here saline was used as the negative control. Aggregation, if any, was detected through phase contrast microscope (Leica DM IRB, Germany) at 20X magnification [1].

### 2.2 Cytotoxicity

In order to test the cellular response, *in vitro* cytotoxicity of the nanoparticles was evaluated on mouse fibroblast cell lines (L929) using MTT assay as per ISO 10993–5:2009 standard. The cells were cultured in MEM medium supplemented with 10% fetal bovine serum and kept at 37 °C in a humidified atmosphere of 5% CO_2_. Cells were seeded in 24 well plates at a density of 0.5 ×10^5^ cells per well and incubated for 24 h with 25, 50, 100 and 200 mM concentrations of nanoparticles. The medium containing test material was removed and MTT was added to each well at a concentration of 100 µg/well and the well plates were incubated at 37 °C for 4 h. The absorbance was measured using a plate reader (Finstruments Microplate Reader USA). Percentage viability was calculated from the ratio of absorbance of the test sample to the absorbance of negative control. The cells cultured in MEM medium with 10% FBS was used as negative control.

### 2.3 Fluorescence Imaging

To check the potential of the Cdts for imaging applications, the fluorescence imaging efficacy of P@ZVI-Cdts was tested for different concentrations (50, 100, 200, 250 and 300 mM) using an optical imaging system (Fig. 4). Concentration dependent variation in the photoluminescence was observed at an excitation wavelength of 540 nm and the emission at 640 nm [2]. Lower energy wavelengths were chosen to suit the imaging applications to avoid interference from autofluorescence while considering *in vivo* imaging [2], [3], [4].

### 2.4 *In vivo* Magnetic Resonance Angiography

Wistar rats weighing 200–250 g were used for MR angiographic studies using C@ZVI nanoparticles. The institutional ethics committee approved the entire animal experiments (No.SCT/ABS/IAEC-83/11 dated 21.02.2014). MRA was performed by using a 1.5 T MRI scanner (Magnatom Avanto; Siemens, Munich, Germany) equipped with a 12 channel head and neck coil. Animals were anesthetized with ketamine (100 mg/kg), xylazine (7 mg/kg) and atropine (0.02 mg/kg) and imaged before and after intravenous tail vein administration of the C@ZVI nanoparticles. After running the routine MRI sequences for acquiring the localizer and T1 weighted sequence, MR angiogram was performed using a 3D-FLASH dynamic contrast-enhanced MRA sequence. Imaging parameters for 3D-FLASH were a TR of 9 ms, a TE of 3.27 ms, a 30° flip angle, a 256 ×232 matrix 250 mm FOV, and a single slab with 17 sections of 1.00-mm effective thickness and 20% interslice gap. The MR angiography was obtained immediately after the bolus intravenous administration of (2.83 mg Fe/kg) iron nanoparticles followed by a saline flush. Color coded image of MRA of C@ZVI administrated animal along with the dorsal and lateral view are shown in Fig. 5. and Fig. 6, respectively [5], [6].
